# The Free-Booter Again

**Published:** 1893-04

**Authors:** 


THE FREE-BOOTER AGAIN.—We have complained several times of the St. Louis *Weekly Review* for piracy on our editorial columns, but the pachydermatous party who hires to Chambers & Co. to do the alleged editorial work on that publication, (it seems to consist mostly of clippings without credit, and appropriating, bodily, editorial matter from its exchanges,) is as insensible to the rights of others as he is regardless of the ordinary courtesies of the profession. He played a shabby trick on us recently. In our February number we had a paragraph (under the head of "A Mere Trifle,") about "a Texas Contemporary," who had said a certain rediculous thing, and we indulged in what was intended for a witticism; it was, at least a *criticism.* The pachyderm who poses as "editor" of the St. Lous Ishmael, the *Weekly Review* with his usual cool effrontery, reproduces the paragraph as original matter, and inserts the name of *Daniel's Texas Medical Journal* in lieu of that "Texas Contemporary" to which we had referred; thus, making us the butt of our own criticism, while he gets credit for the witticism as original. In response to a courteous remonstrance, the *pachyderm* writes us an offensive letter, in which he says "you may be tired of Journals clipping from you without credit, but is a deplorable *custom* which has been inaugurated and followed, and is really not worthy of having attention bestowed upon it;"—claims the *right* to steal editoral matter. We know of no reputable Journalist who is guilty of any such custom. Is there no protection against the wild and wooly Bedouins of the Journalistic field?

We have in Texas a pachydermatous creature, of the genus *sus*, species, *razor-back.* Ravenous for food he eats everything that comes in his way, without a thought or a care as to rights of property. The *Review* reminds us ot him; but we have no standard of comparison by which to measure the utter disregard of the amenities of Journalism and ordinary professional courtesy which has of late characterized the editorial management of that publication.

